# Text summarization method of argumentative discourse by combining the BERT-transformer model

**DOI:** 10.3389/frai.2025.1654496

**Published:** 2025-11-28

**Authors:** Yaser Altameemi, Mohammed Altamimi, Adel Alkhalil, Diaa Uliyan, Romany F. Mansour

**Affiliations:** 1 Department of English, College of Arts and Literature, University of Ha’il, Ha’il, Saudi Arabia; 2 Department of Information and Computer Science, College of Computer Science and Engineering, University of Ha'il, Ha'il, Saudi Arabia; 3 Department of Software Engineering, College of Computer Science and Engineering, University of Ha'il, Ha'il, Saudi Arabia; 4 Department of Information Security, College of Computer Science and Engineering, University of Ha'il, Ha’il, Saudi Arabia; 5 Department of Mathematics, Faculty of Science, New Valley University, El-Kharga, Egypt

**Keywords:** extractive text summarization, abstractive text summarization, bidirectional encoder representations from transformers (BERT), transformer model, argumentative discourse

## Abstract

Summarization of texts have been considered as essential practice nowadays with the careful presentation of the main ideas of a text. The current study aims to provide a methodology of summarizing complex texts such as argumentative discourse. Extractive and abstractive summarization techniques have recently gained significant attention. Each has its own limitations that reduce efficiency in the coverage of the main points of the summary, but by combining them, we can use the positive points of each to improve both summarization performance and summary generation quality. This paper presents a novel extractive-abstractive text summarization method that ensures coverage of the main points of the entire text. It is based on combining Bidirectional Encoder Representations from Transformers (BERT) and transfer learning. Using a dataset comprising two UK parliamentary debates, the study shows that the proposed method effectively summarizes the main points. Comparing extractive and abstractive summarization, the experiment used Recall-Oriented Understudy for Gisting Evaluation (ROUGE) sets of metrics and achieved scores of 30.1, 9.60, and 27.9 for the first debate, and 36.2, 11.80, and 31.5 for the second, using ROUGE-1, ROUGE-2, and ROUGE-L metrics, respectively.

## 1 Introduction

Over the past two decades, a vast and wide range of data has become available on the internet, such as articles, tweets, and news. This huge amount of data presents problems for many specialists, such as journalists, politicians, and researchers, who need to extract the main points through a process of summarization (Al Qassem et al., 2017). Recently several studies have applied applications that provide the summarization features of texts (e.g., Abualigah et al., 2020; El-Kassas et al., 2021; Widyassari et al., 2022). Although there is a rapid development in text summarization specifically within the era of AI (e.g., Dar et al., 2024; Das and Mohapatro, 2025; Gera et al., 2022; Shakil et al., 2024; Zhang et al., 2023), specific features are needed in the text summarization which requires deep development of text summarization model.

Three general considerations can be applied to the summarization process. The first is the type of input or source from which the summary is to be extracted; it can be single- or multi-document. The second is the context, which is classified as generic (using the original text to obtain the context), query-driven (important information is provided by the user), or domain-specific (with domain knowledge to help extract the summary). For more information about summarization based on context, see Sarkar (2009). The third consideration is the output type, which can be either extractive or abstractive summarization. In the extractive process, the summary is extracted from the main documents based on statistical and linguistic characteristics (Mihalcea and Tarau, 2004). In the abstractive process, the summarization is based on applying various words that depend on the real semantics of the document (Al Qassem et al., 2017; Sarkar, 2009). Abstractive summarization is complex, using natural language processing (NLP), machine learning techniques, and, more recently, deep learning models to facilitate semantic analysis (Azmi and Altmami, 2018; Wazery et al., 2022).

The merits of abstractive and extractive summarization are hotly debated. Those who support extractive summarization believe that it provides salient sentences from a given text, giving straighter and more robust results than its rival (Mao et al., 2019). On the other hand, students of abstractive summarization argue that it might be better in terms of cohesion and readability (Kouris et al., 2022), with the output resembling summaries generated by humans because it contains rephrased sentences with new words. Both methods have effective tools for summarizing texts, so combining them may overcome the obstacles preventing high-performance summarization. Using one technique over the other might cause unstructured sentences (this point will be discussed in more detail in the following section). Therefore, this research’s first contribution is to propose a new methodology combining both extractive and abstractive text summarization methods, thus increasing the performance of text summary as measured using ROUGE metrics.

This paper also highlights the importance of considering the theoretical and philosophical views of linguistic structure. The authors suggest that much research in the summarization area has focused on increasing efficiency numerically, through calculations, without considering the abstractive nature of language, such as semantic and pragmatic structures. Our study’s second contribution relates the summarization technique to the theoretical nature of language. We follow Fairclough and Fairclough (2012), who suggested the importance of considering the genre of discourse before analyzing it. The current case involves very complex parliamentary debates in which speakers contend among themselves to support specific claims. Altameemi (2020) highlighted the difficulty of analyzing the whole of a debate by analyzing the speeches of its key speakers. He argued that analyzing the whole debate is effective, but it takes ages to do so manually. Therefore, this paper aims to fill this gap by summarizing the main elements of arguments that help present the overall picture of the arguments made in a parliamentary debate.

In this paper, the authors first present relevant works that focus on abstractive and extractive summarization. These methods are discussed in relation to summarizing the specific genre of text, political discourse, because the nature of this complex genre, with its argumentative structure, makes it an issue for many analysts. Next, we discuss the proposed model for conducting the experiments, including the training dataset, evaluation metrics, and experimental setting. We then discuss the results of the experiments and show how the proposed model—applying extractive and abstractive summarization in the same sequence—has filled the gap of increasing the efficiency of political discourse summary.

## 2 Related work

A wide range of summarizations has been carried out using both abstractive and extractive text methods (Gambhir and Gupta, 2017). Both techniques provided high-quality results. This section reviews recent publications related to summarizing argumentative texts.

Extractive summarization is based on ranking the importance of each sentence and then returning the first few sentences with the highest rank as main sentences (Gupta and Lehal, 2010). It involves three basic steps: text preprocessing, sentence ranking, and sentence selection (Ferreira et al., 2013). The earliest approaches to automatic summarization focused on extractive techniques, starting by determining the importance of each sentence according to its similarity score. Each sentence of the input article is scored, and those with the highest scores are ranked in the summary. Early extraction summarization methods include TextRank (Ashari and Riasetiawan, 2017; Mihalcea and Tarau, 2004), a graph-based technique that ranks sentences according to the key score of each (Erkan and Radev, 2004; Parveen et al., 2015).

Later, recent developments in computer hardware and software, such as machine learning, were incorporated to identify the similarity score of each sentence. For instance, John and Wilscy (2013) summarized multidocuments from the Document Understanding Conferences(DUC) dataset using a random forest classifier. Their approach was based on classifying the sentences with the highest relevance with respect to the rest of the sentences generated for the summary. Using the same dataset, Fattah (2014) employed maximum entropy, naive Bayes, and support vector machine models to summarize multidocuments. Generally, machine learning methods achieved substantial results in the text summarization domain. However, at some point, the efficiency of the learning process started to suffer from the limited sizes of datasets; it could not compete with graph-based models (Yadav et al., 2022).

Models based on neural networks overcame the limitations of those based on machine learning and produced even better results than graph-based models. For example, Yousefi-Azar and Hamey (2017) implemented Seq2seq and encoder-decoder based models for extractive text summarization and Nallapati et al. (2016a) used Recurrent Neural Networks (RNNs) for text summarization selection.

Transformer models have been applied using neural network architecture designed for natural language processing. Roush and Balaji (2020) fine-tuned several transformer models for word-level extractive summarization. The experiments were performed using the DebateSum dataset, which consists of 187,386 unique pieces of evidence with corresponding arguments. They evaluated their experiments with ROUGE metrics, achieving scores of 56.32 ROUGE-1 using Bidirectional Encoder Representations from Transformers BERT-Large (developed by Devlin et al., 2018), 52.07 ROUGE-1 using Generative Pre-trained Transformer GPT2-Medium (produced by Radford et al., 2019), and 60.21 ROUGE-1 using Longformer-Base-4096 (designed by Beltagy et al., 2020). They observed that the Longformer model achieved the best results because of its long-range context, which helped in choosing the tokens to be included in the summary.

Duan et al. (2019) performed text summarization of a civil trial debate involving many participants—the plaintiff, defendant, witnesses, and judge, for example. They performed several baseline experiments using the TextRank model, an RNN based on a sequence model, Long Short-Term Memory (LSTM), and Transformer. They achieved their best score, 34.8 ROUGE-1, using Transformer. They compared this result with their own method of using utterances, achieving best scores of 19.9 ROUGE-2 and 36.18 ROUGE-L.

Alshomary et al. (2021) performed contrastive learning via a Siamese neural network to match arguments to key points before applying a graph-based extractive summarization model to generate key points. Their experiments used a dataset containing 6,515 arguments and 243 key points. They achieved their best summarization results using the graph-based approach, achieving a score of 19.8 ROUGE-1.

Unlike extractive summarization, abstractive summarization is based on paraphrasing the main content of a document using novel words that might not exist in the original document (Nallapati et al., 2016a). Recently, numerous studies have used this method instead of extractive summarization. Chowanda et al. (2017) applied abstractive summarization using the point-based summarization technique. This relies on extracting the main points’ verbs before extracting the main point itself based on the dependency parse and syntactic frame. They achived a ROUGE-1 score 8.99% higher than that achieved by point-based summarization.

Wazery et al. (2022) implemented abstractive text summarization based on a sequence-to-sequence model, using several deep learning models. They investigated different layers of Gated Recurrent Units (GRUs), Long Short-Term Memory (LSTM), and Bidirectional Long Short-Term Memory (BiLSTM). They performed their experiments on two datasets using the Arabic language: the Arabic Headline Summary (AHS) and Arabic Mogalad_Ndeef (AMN) datasets. They achieved their best results with BiLSTM, achieving consecutive scores of 51.49 and 44.28 ROUGE-1.

Chen and Yang (2021) applied a structure-aware sequence-to-sequence model by combining the discourse relations between conversations with the connections between speakers and actions within each conversation. They conducted their experiments on conversation levels using the SAMSum corpus training set, containing 14,732 dialogs, and the 819 dialogs of testing set (Gliwa et al., 2019). Their method’s best results achieved a score of 46.07 ROUGE-1. In comparison, Lewis et al. (2019), using BERT, achieved a score of 45.15 ROUGE-1.

Using one technique rather than the other causes an issue with summarization. For example, abstractive summarization has attracted recent researchers because it replaces text summarization by coming up with new words or phrases. However, it does not produce grammatically structured sentences, although the output does look as if it had been written by a human. Similarly, extractive summarization often suffers from inadequate or incorrect content, largely due to the unstructured and complex characteristics of human interactions (Chen and Yang, 2021). Extractive techniques can extract the most relevant information or sentences, but they do not produce the fluency and coherency between sentences that would be expected in a summarization generated by a human (Pilault et al., 2020). Extractive techniques retain their attraction because they are computationally cheaper and generate grammatically and semantically correct summaries most of the time (Nallapati et al., 2016b).

Although the two techniques show good results and clear development in summarizing texts, the previous studies ignored the nature of the argumentation discourse to some extent. In this research, we focus on summarizing ideas arising from parliamentary discourse. Many researchers (Baker et al., 2008; Chilton, 2004; Forchtner and Tominc, 2012; Kwon et al., 2009; Trimithiotis, 2018; Wodak, 2009) have recommended that analysts of discourse consider the genre/type of the text when specifying the analysis methodology. Many research articles (Chen and Song, 2021; Fattah, 2014; Sarkar, 2009; Yousefi-Azar and Hamey, 2017) have focused on summarization techniques without paying much attention to the type of text. We consider this issue here by looking at how techniques can participate in summarizing argumentation discourse and, more specifically, parliamentary discourse. This allows us to consider the importance of aligning the applied techniques with the nature of the discourse.

The structure of a parliamentary discourse differs from that of other discourses because its entire structure hinges on the main arguments of the speakers. In addition, ideas move forward and backward as different Members of Parliament (MPs) intervene. Although the interventions are managed and controlled by the Speaker, the ideas presented become subjects for debate themselves. Another point is that during the summarization process for this current project, we focused on specifying the main ideas and concepts discussed by the speakers rather than on the validity of their arguments. This enables us to isolate the central points of the overall debate, even before looking at the debate itself. Filling this gap will help analysts decrease the bias on specifying the ideas represented in the debate that are of more concern than others.

Altameemi (2020) analyzed the speeches of the Prime Minister and the Leader of the Opposition in detail. He suggested summarizing whole debates before analyzing the speeches of the leaders to gain a deep understanding of the specific contexts regarding the salient ideas debated by MPs. However, the manual summarization presented an issue because each debate consisted of around 70,000 words. Therefore, in this study, we try to fill this gap as well as increase the summarization efficiency by using a hybrid method.

A central gap arises from the four common features of human summarization (Pilault et al., 2020) and we have considered three of these in the motivation of our current research. First, humans can infer the original context. Second, they can order the most important parts of the text. Third, they can organize the summary of the most important ideas into relevant sentences. Extractive summarization helps tackle the first and second points, while abstractive summarization helps tackle the third. Having tried to mimic human text summarization by combining all three features, we wanted to combine extractive and abstractive text summarization.

## 3 Proposed system

The proposed approach in the current study was developed after considering the importance of the improvements that could be made by combining the two techniques. The use of the extractive summarization technique in the first phase ensures that the most important parts of the text are selected and that the selected sentences are grammatically written and structured. In the second phase, abstractive text summarization is applied to the summarized text from the first phase. The combination of the two techniques is intended to ensure that the texts are modified to appear as a summary created by a human.

**Figure 1:**
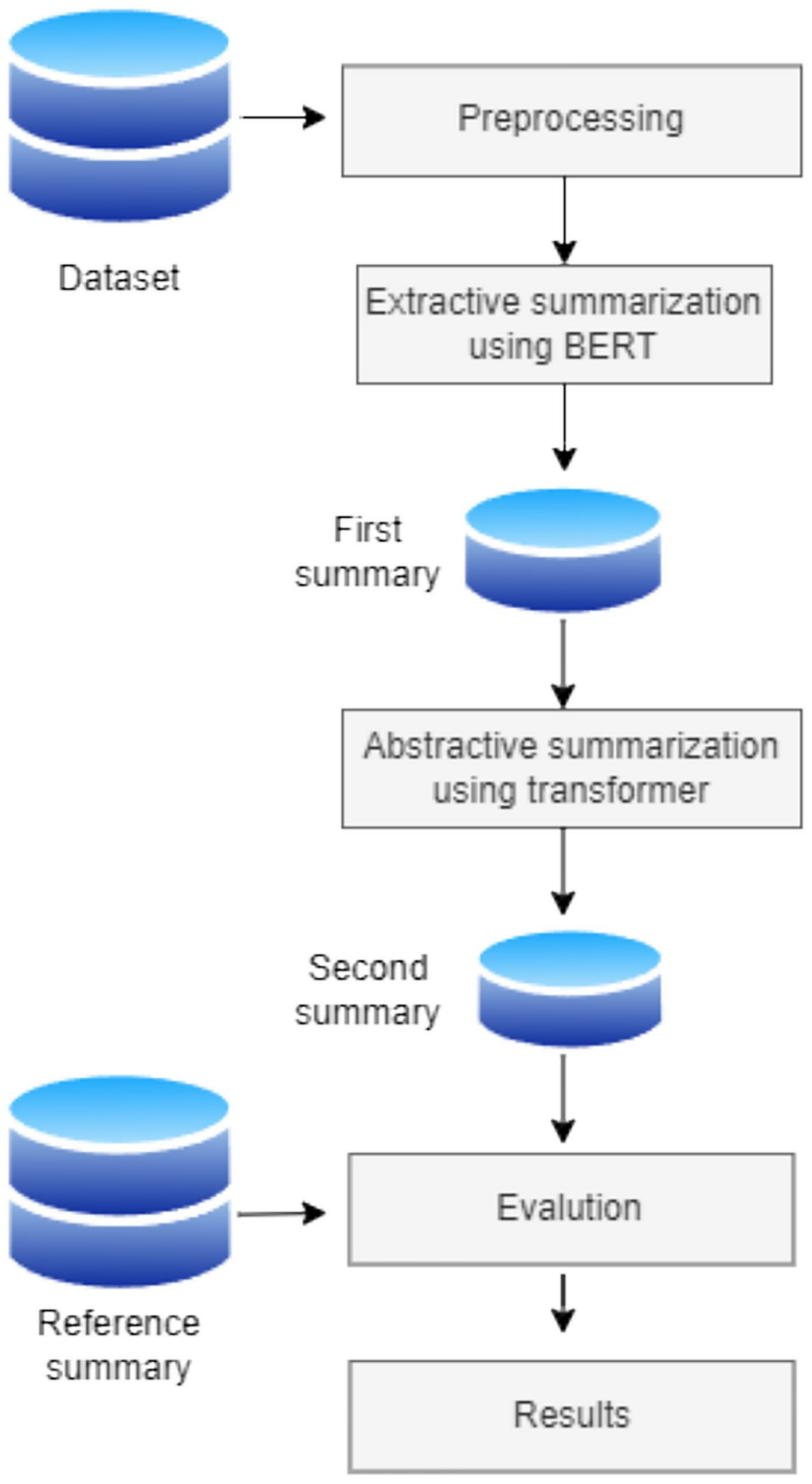
Framework for text summarization.

The system followed in this study consists of four phases: data preprocessing, extractive summarization, abstractive summarization, and model evaluation. In addition, the system includes two data sources: the original debate dataset and a reference summary. Figure 1 illustrates the process framework.

### 3.1 Datasets

The experiments were implemented using the debate context. The dataset consisted of two UK parliamentary debates at two different times. The first debate was held on August 29, 2013, a week after the Syrian government’s use of chemical weapons against rebels. In that debate, David Cameron (the Prime Minister) called upon MPs to support a possible UK military action against the use of chemical attacks in Syria. At the time of the debate, the details of the attack were unclear, and evidence of the use of chemical weapons had not been validated. The majority of MPs voted not to support any possible action by the UK until the United Nations issued a resolution. The second debate, on December 2, 2015, concerned the expansion of military action against Islamic State of Iraq and the Levant (ISIL)^1^, a terrorist group, from Iraq to Syria. In this second debate, there was a salient shift, as the majority of MPs supported the extension of airstrikes from Iraq to Syria. Both debates were used for the training dataset, as shown in Table 1.

**Table 1:** Statistics of our training dataset.

Selected corpus	Number of interventions by speakers in parliament	Number of words
Debate-1	502	67,655
Debate-2	759	95,847

For our testing dataset, we adopted manual summarization of both debates, basing it on analysis of the main points mentioned by Altameemi (2020), who summarized the main ideas of the arguments in his analyses. We used this as our reference summary.

Before starting the experiment, we divided the dataset text into chunks, trying various numbers of chunks. We found that longer sequences of chunks gave us less representative summarization.

### 3.2 Experiment setup

We applied three summarization techniques: extractive, abstractive, and a combination of the two.

For extractive summarization, we employed the BERT model, using the Bert-Extractive-Summarizer library (Miller, 2019). The model works by embedding the sentences and then using a clustering technique involving a k-means clustering algorithm to identify the sentences closest to the centroid for summary selection. We used BERT extractive summarization to generate the first summarization and extract the most representative sentences of the article. We generated a maximum of three sentences for each chunk to represent the closest sentences for the summary.

For abstractive summarization, we used transfer learning with a transformer model. We employed a pre-trained model for specific summarization, which obviated the need for a large set of labeled data, using Bart-Large-CNN, a transformer library pre-trained in the English language, and the CNN/Daily Mail dataset (Lewis et al., 2019).

We used the transformer to generate the second summarization and produce sentences summarizing the content of the first summarization task. For this phase, we generated a summary that had a maximum of 50 words for each chunk.

### 3.3 Evaluation metrics

Currently, ROUGE, presented by Lin (2004b), is the most used set of evaluation metrics in the field of text summarization. The package we chose, introduced by Lin (2004a), is used to compare results and measure the qualities of summarization models. It does this by calculating the overlap between the summary generated by the model and the human-generated reference summary. A ROUGE-1 score refers to the percentage of overlap between the generated and reference summaries in terms of each word (unigram), a ROUGE-2 score refers to the percentage in terms of two connected words (bigram), and a ROUGE-L score refers to the percentage in terms of sentence level, using the longest common subsequence. In this paper, we used the F-measure of recall and precision of each ROUGE-1, ROUGE-2, and ROUGE-L score.

## 4 Results and analyses

Our first abstractive summarization, using Debate-1, achieved scores of 0.298 ROUGE-1, 0.105 ROUGE-2, and 0.278 ROUGE-L. Our next summarization was extractive, using the same dataset, and achieved scores of 0.309 ROUGE-1, 0.099 ROUGE-2, and 0.288 ROUGE-L. For the third trial with this dataset, we first performed an extractive summarization by transformer, sending the summarized text to the abstractive BART layer to generate the final summary. This method achieved higher ROUGE-1 (0.301) and ROUGE-L scores (0.279) than abstractive summarization alone, although its ROUGE-2 score was slightly lower, at 0.096.

We went on to run a set of additional comparative experiments using the same methodologies but on a different dataset, Debate-2. The combined summarization technique also achieved better results than abstractive summarization alone, achieving scores of 0.362 ROUGE-1 and 0.315 ROUGE-L.

Table 2 shows the results of all the experiments conducted for the two parliamentary debates.

**Table 2:** Summarization results of all three experiments using ROUGE score.

Metrics	Abstraction transformer		Extraction BERT		Proposed method Extraction then abstraction	
	Debate-1	Debate-2	Debate-1	Debate-2	Debate-1	Debate-2
ROUGE-1	0.298	0.354	0.309	0.360	0.301	0.362
ROUGE-2	0.105	0.143	0.099	0.123	0.096	0.118
ROUGE-L	0.278	0.311	0.288	0.314	0.279	0.315

Although the extraction with BERT scored more highly than the proposed method did, the latter created summaries whose semantic structure appears to be more efficient than that of those produced by extraction alone, which offered many unimportant clauses, as shown in the examples below.

Example 1:

*‘Will the Prime Minister give way on that point? The Prime Minister.’*

Although this statement features heavily, marking speakers’ interventions, it is not as important as the main ideas in the debate.

Abstraction appears to be more coherent than extraction, as Example 2 shows.

Example 2:

*The Prime Minister says there is no 100% certainty about who is responsible for the attack in Syria. He says the biggest danger of escalation is if the world community stands back and does nothing.*

This example shows how the abstraction method not only uses the exact words in the original text but also paraphrases them, for example by using pronouns to report parts of Cameron’s speech instead of placing his name at the start of each of his utterances, as in the extraction.

Although abstraction is effective in the paraphrasing process, issues still appear with regard to the semantic structure, as Example 3 shows.

Example 3:

*I have not yet heard a compelling argument to convince me that military intervention. There are many compelling arguments for doing nothing.*

The missing verb in the first sentence shows that the idea of convincing MPs is not complete, although the reader can predict the whole idea from the context. Although the efficiency of the abstractive analysis score has scored well, we argue that some ideas are not fully covered in the summarization. We therefore decided to apply extractive summarization first because it focuses on the content, and then apply abstractive summarization because it considers the importance of focusing on the meaning of the main text. The value of this approach appeared clearly in the combination of the two techniques, as Example 4 shows:

Example 4:

*Legal experts are saying that without explicit UN Security Council reinforcement. Especially when so many legal experts say that without explicitly UN Security Council reinforcement.*

The idea of legal experts appeared in the extraction, which is the first step. It was then presented in the context of the semantic structure by linking it to the role of the UN inspectors. This shows that the proposed method fixed issues in the summarization process by utilizing the positive features of each technique.

Analysis of the second debate produced similar findings. First, we have extraction, which focuses on the frequency of the words and on summarizing the whole text into various disconnected chunks, as shown in Example 5.

Example 5:

*It is certainly true that there have been well documented cases of such weapons ending up in the hands of Daesh. I think that changes need to be made to the Government approach. While it is all very well metaphorically to stand alongside our allies, the very destruction of the caliphate state is in itself the right thing to do. Nor the argument that the Government are proposing the indiscriminate bombing of Syrians. We do not have the ground forces in Syria that I believe we should have.*

It is clear here that coherence between sentences is an issue and that the presentation does not cover the broad context of the whole text. In other words, the extraction technique jumps between ideas in some cases, even though the overall summarization covers the central ideas debated.

However, as mentioned in the analysis of Debate-1, the central issue with extraction is the focus on covering the content through the frequency of the words. Abstraction produces better semantic representation, for example of the subject of fighting terrorism. The extraction technique refers to terrorism in the passage “we do not have the migration crisis and we do not have the terrorism crisis,” while abstraction produces clearer representation that relates more to the contested arguments, as shown in Example 6.

Example 6:

*I have made my views clear about the importance of all of us fighting terrorism and I think that it is time to move on.*

The summary here shows the importance of fighting ISIL to counter terrorism, which is a threat to national and international security. Abstractive summarization makes a clearer connection between fighting terrorism and national security than does extractive summarization.

The proposed method in this study began with the extractive technique. When we then applied the abstractive technique, the summary included text like that shown in Example 7.

Example 7:

*So, I urge those who say that air strikes would increase that danger not to give into that narrative. These people are already targeting us now.*

Here, ISIL’s threat to the British people and the fight against terrorism are represented as central ideas and concepts in the debate. According to Altameemi (2020), the Government’s main claim in the debate is the need for urgent action against ISIL because this terrorist group threatens the British community. Our proposed method linked “terrorism” to the main debated claim in Parliament. Therefore, the combination of abstractive and extractive methods increased summarization efficiency by linking the summarized ideas to the main points of the debate. This helps linguistic analysts obtain the central contested ideas in a long parliamentary debate. Applying this method would help automatic summarization achieve a fuller understanding of the parliamentary context for any specific topic.

## 5 Conclusion and future work

In this paper, we introduce a two-phase combination of extractive and abstractive summarization techniques for parliamentary discourse. The first step is to preprocess the text by breaking the input document into chunks. This is followed by the first phase of extractive summarization for each chunk, ensuring that the main points of the entire debate are covered. In the second phase, the output from the first layer of summarization is passed on for abstractive summarization. This method ensures that the text is summarized and modified to look as if it were written by a human. It also provides better performance than the extractive summarizer alone. Using the Debate-1 dataset, our method was better by 0.003% ROUGE-1 and 0.001% ROUGE-L. Using the Debate-2 dataset, it was better by 0.008% ROUGE-1 and 0.004% ROUGE-L.

For future work, we are considering splitting the dataset, performing different summarization techniques on each portion, and then combining the outcome summary of each technique into one dataset before comparing the results with those accomplished by either abstractive or extractive techniques alone. Chen and Song (2021) have already applied this set of techniques, but their experiment used traditional extractive summarization methods, such as TextRank.

## Data Availability

The original contributions presented in the study are included in the article/supplementary material, further inquiries can be directed to the corresponding author.

## Footnotes

1 This group is also called Daesh as the MPs in the parliamentary debate use both ISIL and Daesh.
